# Comparison of Clinical Efficacy and Safety between 70–150 µm and 100–300 µm Doxorubicin Drug-Eluting Bead Transarterial Chemoembolization for Hepatocellular Carcinoma

**DOI:** 10.3390/life12020297

**Published:** 2022-02-16

**Authors:** Jung Woo Yi, Hyun Pyo Hong, Myung Sub Kim, Byung Seok Shin, Heon-Ju Kwon, Byung Ik Kim, Won Sohn

**Affiliations:** 1 Department of Radiology, Kangbuk Samsung Hospital, School of Medicine, Sungkyunkwan University, Seoul 03181, Korea; yijw8282@gmail.com (J.W.Y.); summersonrad@naver.com (H.P.H.); heonju.kwon@samsung.com (H.-J.K.); 2 Department of Radiology, Chungnam National University Hospital, Daejeon 35015, Korea; starzan@chol.com; 3 Department of Internal Medicine, Kangbuk Samsung Hospital, School of Medicine, Sungkyunkwan University, Seoul 03181, Korea; bik.kim@samsung.com (B.I.K.); wonsohn1@gmail.com (W.S.)

**Keywords:** hepatocellular carcinoma, transcatheter arterial chemoembolization, doxorubicin drug-eluting beads, response, safety

## Abstract

Background: This study aimed to compare the efficacy and safety of 70–150 μm doxorubicin drug-eluting bead (DEB) transarterial chemoembolization (TACE) with those of 100–300 μm DEB-TACE as first-line treatment in patients with hepatocellular carcinoma (HCC). Methods: We retrospectively investigated 72 patients who underwent TACE with 70–150 μm DEBs (*n* = 40) or 100–300 μm DEBs (*n* = 32) for HCC in a tertiary center between March 2013 and May 2019. Initial treatment response and adverse events were assessed using the modified Response Evaluation Criteria in Solid Tumors and the National Cancer Institute Common Terminology Criteria for Adverse Events version 5.0, respectively. Results: At the 2-month post-treatment assessment, the complete and objective response rates were 47.5% and 85.0%, respectively, for the 70–150 μm group and 34.4% and 81.3%, respectively, for the 100–300 μm group; however, the difference was not statistically significant (*p* > 0.05). In total, 65% patients in the 70–150 μm group and 59.4 % patients in the 100-300 μm group experienced at least one symptom of post-embolization syndrome after TACE; all symptoms were classified as grade 1 or 2. There was no significant difference between the two groups in terms of post-procedural laboratory changes such as changes in liver enzymes and bilirubin levels (*p* > 0.05). Laboratory toxicity of grade 3 occurred in three patients, all of which were transient elevation of liver enzyme levels. Hepatobiliary adverse events, such as bile duct injury, biloma, liver abscess, and hepatic infarction, were not observed in either treatment group. Conclusion: This study found no significant difference in tumor response between 70–150 μm and 100–300 μm DEB-TACE. Both groups showed favorable safety profiles, and the difference was not significant.

## 1. Introduction

Hepatocellular carcinoma (HCC) is the third leading cause of cancer-related mortality and the most common primary hepatic malignancy worldwide [1]. Although potentially curative treatments for HCC include liver transplantation, surgical resection, and local ablation, these procedures are available for <30% of patients with early-stage HCC [2]. Transarterial chemoembolization (TACE) is the first-choice treatment in patients with intermediate-stage HCC [3]. Moreover, TACE is performed for the treatment of early stage HCC when resection, ablation, or liver transplantation is not feasible [4]. In conventional TACE (c-TACE), an emulsion of lipiodol and chemotherapy drugs is commonly used to increase the local drug concentration and embolize targeting arteries. However, c-TACE is a heterogeneous technique with no standardized approach, and chemotherapy drugs can easily diffuse into the systemic circulation, resulting in high systemic toxicity [5]. Drug-eluting bead (DEB) TACE involves the use of drug-loaded microspheres to increase the concentration of chemotherapy drugs in the tumor and reduce systemic chemotherapy levels [6]. Several prospective randomized trials comparing c-TACE and DEB-TACE in patients with HCC have reported that DEB-TACE is associated with less liver toxicity and postprocedural pain [7,8]. However, an improvement in tumor response and overall survival with DEB-TACE compared to that with c-TACE has not been confirmed [8,9]. 

In clinical practice, different sizes of DEB particles are available for use. Recent studies indicate that TACE with 100–300 μm DEBs is associated with a higher survival rate, improved tumor response, and lower complications than TACE with 300–500 μm or 500–700 μm DEBs [10,11]. In animal models, 100–300 μm beads were detected primarily within the tumor after embolization, whereas 300–500 μm beads were distributed in the tumor periphery [12]. A new class of DEBs with 70–150 µm beads (DC Bead M1; Biocompatibles UK Ltd., BTG) has been developed recently. In a recent study, 70–150 μm DEB-TACE achieved impressive objective response rates of 94.5% at 3 months, with a median overall survival of 42.0 months [13]. There are a few small retrospective studies that have compared the outcomes of 70–150 μm and 100–300 μm DEB-TACE [14,15,16,17]. However, since these studies have reported different results, it is difficult to confirm whether 70–150 µm DEB-TACE is superior to 100–300 µm DEB-TACE in terms of outcomes in patients with HCC. Moreover, there is a lack of studies comparing outcomes between 70–150 μm and 100–300 μm DEB-TACE as first-line treatment for patients with HCC. Therefore, it is necessary to compare the clinical outcomes between the two types of DEB-TACE in treatment-naïve HCC patients. Our institution has gradually changed the bead size used in TACE from 100–300 μm to 70–150 μm, reflecting the trend toward using smaller beads in DEB-TACE. Thus, this retrospective study aimed to compare the efficacy and safety of 70–150 μm with those of 100–300 μm DEB-TACE used as the first-line treatment in patients with treatment-naïve HCC.

## 2. Materials and Methods

### 2.1. Patient Population

**Figure 1:**
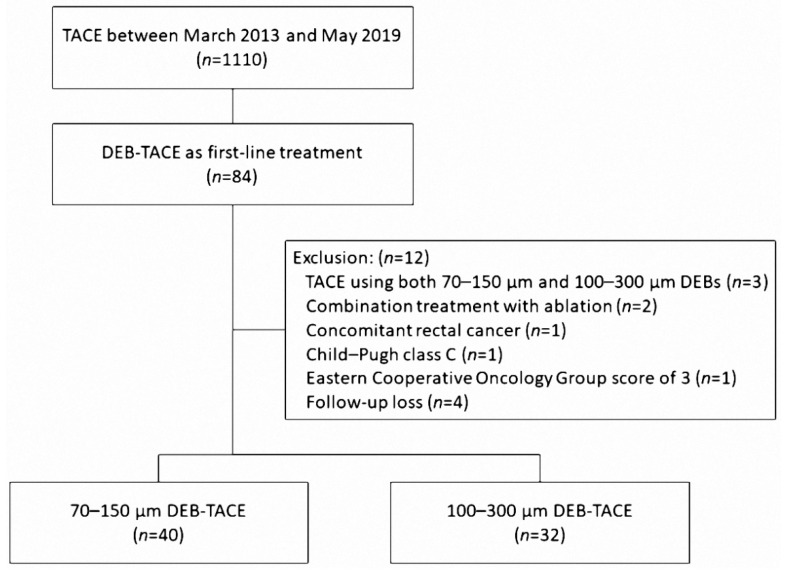
Flowchart of patient selection.

Our institutional review board approved this study and waived the requirement for patient consent because of the retrospective nature of the review. HCC was diagnosed according to the American Association for the Study of Liver Diseases criteria. In our institution, TACE is indicated when hepatic resection cannot be performed due to advanced stage or insufficient hepatic reserve or when ablation therapy is not indicated because the tumor size is larger than 3 cm and there are multiple lesions (more than three), vascular invasion, or technical contraindications (invisible on planning ultrasound for ablation or risk of thermal injury to adjacent organs). Patients who underwent DEB-TACE as first-line treatment were included in this study. Patients who previously underwent surgical resection, ablation therapy, or TACE were excluded because previous treatments might have introduced bias into the evaluation of the effectiveness of DEB-TACE. Between March 2013 and May 2019, DEB-TACE was performed on 84 patients as first-line treatment. A total of 12 patients were excluded for the following reasons: TACE using both 70–150 μm and 100–300 μm DEBs (*n* = 3), combination treatment with ablation (*n* = 2), concomitant rectal cancer (*n* = 1), Child–Pugh class C (*n* = 1), Eastern Cooperative Oncology Group score of 3 (*n* = 1), and follow-up loss (*n* = 4). Finally, a total of 72 patients were enrolled in our study, including 40 treated with 70–150 μm DEBs and 32 treated with 100–300 μm DEBs (Figure 1). Since 70–150 µm DEBs were available in Korea from 2017, DEB sizes of 100–300 μm were used from March 2013 to late 2016. Thereafter, the 70–150 μm DEB sizes were used until May 2019.

### 2.2. DEB-TACE Procedure

**Figure 2:**
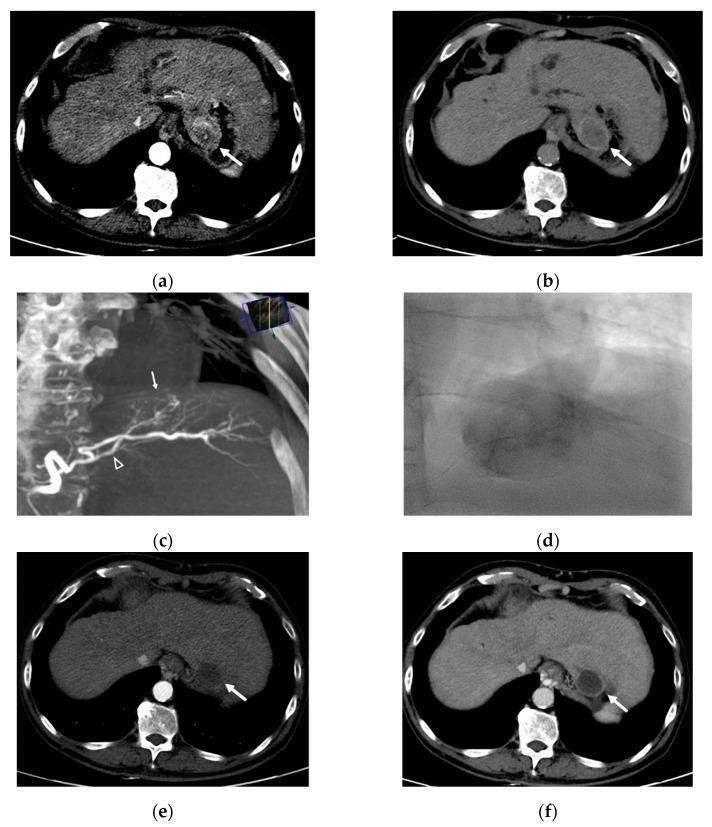
A 61 year-old man with HCC. (**a**) Arterial phase of dynamic CT shows a hypervascular mass measuring 4.1cm in diameter in segment 2 (arrow). (**b**) Delayed phase image from the same study shows classic tumor washout (arrow). (**c**) Cone-beam CT angiography shows a tumor blush (arrow) and precisely delineates single tumor feeder (arrowhead). (**d**) After selection of tumor feeder using microcatheter, DC Bead (70–150 µm) loaded with doxorubicin/nonionic contrast suspension is slowly injected. (**e**,**f**) Arterial and delayed-phase CT scan 3 months later shows no enhancement in the tumor, indicating complete response.

All procedures were performed by one interventional radiologist with 10 years of experience. After common femoral artery cannulation under local anesthesia, diagnostic angiography of the superior mesenteric artery and celiac artery (including cone-beam computed tomography [CT]) was performed with a 5-Fr catheter (Yashiro; Terumo, Tokyo, Japan) to outline the anatomy and delineate the tumors. A 2.0-Fr coaxial microcatheter (Progreat, Terumo) was then used to catheterize the feeding vessels supplying the HCC. The microcatheter was placed as distally as possible into the vessel supplying the tumor, and the tip of the catheter was advanced into the hepatic artery and the feeding branch if the size, location, and blood supply were allowed. Under fluoroscopy, each feeding vessel to the tumor was embolized by slow injection of an iodinated contrast material mixed with either 70–150 μm or 100–300 μm DC beads (Biocompatibles UK Ltd., Farnham, UK, BTG) impregnated with 50 mg of doxorubicin in each vial. The doxorubicin dose was calculated according to the infused microsphere volume. For example, when half of one vial of DEB was infused, the used doxorubicin dose was calculated as 25 mg. Termination of injection was indicated by stagnant flow in the feeding hepatic arteries of the tumor. If the embolization endpoint was not achieved after injection of the scheduled volume of loaded beads, polyvinyl alcohol (PVA) particles or gelatin sponge particles were administered until near stasis of the target lesion had been reached. A representative case is shown in Figure 2.

### 2.3. Follow-Up and Assessments

Regular clinical follow-up was performed with laboratory and imaging studies. The first follow-up outpatient visit with dynamic contrast-enhanced CT was generally performed after a 2-month interval (range, 1–3 months). Tumor response was classified according to the modified Response Evaluation Criteria in Solid Tumors (mRECIST) [18]. With the mRECIST criteria, a complete response (CR) was defined as the disappearance of any intra-tumoral arterial enhancement in all lesions; a partial response (PR) was defined as a >30% decrease in the sum of the diameters of viable lesions; progressive disease (PD) was defined as an increase of >20% in the sum of diameters of the viable lesions; and stable disease (SD) was defined as any response that did not qualify as either PR or PD. The objective response rate was defined as the summation of CR and PR. At the first follow-up visit, the subsequent management plan was decided by multidisciplinary teams, depending on the patient’s general condition, laboratory findings, and tumor response evaluation on CT scan.

Adverse events and laboratory test assessments were based on the Common Terminology Criteria for Adverse Events (CTCAE), version 5.0 [19]. All medical events considered to be related to the procedure and the disease were recorded, including any symptoms or signs. The presence of post-embolization syndrome (PES) was assessed during the post-procedural hospital stay. Discharge was delayed if any adverse events requiring major medical attention or therapy occurred. Laboratory test results were recorded for analysis within 2 weeks before the procedure, within 5 days after the procedure (as the worst value in case of multiple tests), and at the first follow-up after the procedure. Hepatobiliary complications, such as biloma, liver abscess, hepatic infarction or portal vein thrombosis were also evaluated. Serious adverse events were defined as any event resulting in death, any immediate life-threatening condition, unscheduled hospital visit or prolonged hospitalization, or permanent or significant disability or incapacity. Prolonged hospitalization was defined as that lasting for more than 7 days.

### 2.4. Statistical Analysis

All statistical analyses were conducted using SPSS software (version 22; SPSS, IBM Corp., Armonk, NY, USA). Continuous variables were compared between groups using Student’s *t*-test (or the Mann–Whitney U test, if appropriate), and categorical variables were compared between groups using the chi-square test (or Fisher’s exact test, if appropriate). Patient characteristics including interventional and clinical data associated with the complete response were identified. Statistical significance was set at *p* < 0.05.

## 3. Results

### 3.1. Baseline Characteristics

Both groups were comparable in terms of baseline characteristics, tumor characteristics, and DEB-TACE (Table 1 and Table 2). Baseline patient characteristics such as age, sex, Child–Pugh, and Barcelona Clinic Liver Cancer (BCLC) stage were similar between the 70–150 μm and 100–300 μm DEB groups (Table 1). The maximum tumor diameter in the 70–150 μm group (mean, 3.57 ± 2.2 cm) was higher than that in the 100-300 μm group (mean, 2.99 ± 1.47 cm), but the difference was not statistically significant (*p* = 0.185) (Table 2).

### 3.2. Treatment Response

The treatment response is summarized in Table 3. At the first follow-up after DEB-TACE, of the 40 patients receiving 70–150 μm DEB-TACE, 19 (47.5%) showed CR, 15 (37.5%) showed PR, four (10.0%) showed SD, and two (5.0%) showed PD. Of the 32 patients who received 100–300 μm DEB-TACE, 11 (34.4%) showed CR, 15 (46.9%) showed PR, five (15.6%) showed SD, and one (3.1%) showed PD. The CR rate was higher in patients treated with 70–150 μm DEB-TACE than in those treated with 100–300 μm DEB-TACE. However, this difference did not reach statistical significance (*p* = 0.262). In addition, there was no statistically significant difference in the objective response rate between the two groups (34 (85.0%) vs. 26 (81.3%), *p* = 0.671). The maximum tumor diameter and the sum of the tumor diameters were identified as factors affecting CR (*p* = 0.011 and *p* = 0.004, respectively). No other factors were shown to be associated with the CR (Table 4).

### 3.3. Safety Assessment

The incidences of clinically symptomatic adverse events are summarized in Table 5. Clinically symptomatic adverse events occurred in 62.5% (45/72) patients; 65% (26/40) in the 70–150 μm group and 59.4% (19/32) in the 100–300 μm group. The adverse events included fever (*n* = 32), abdominal pain (*n* = 31), and vomiting (*n* = 5). All symptoms were mild and classified as grade 1 or 2; no grade 3 or 4 adverse events were recorded. The symptoms subsided within 2–3 days after being managed conservatively. There were no statistically significant differences in terms of adverse events between the two groups. Five patients experienced prolonged hospitalization (≥7 days) due to persistent grade 1 fever. Hepatobiliary adverse events, such as bile duct injury, biloma, liver abscess, and hepatic infarction, were not observed in either treatment group.

Table 6 shows the differences between the laboratory data before and within 5 days of DEB-TACE. Serum liver enzymes and bilirubin levels were elevated after DEB-TACE. However, at the first follow-up visit, the findings were similar to those of the baseline assessment. Changes in laboratory values between the two groups did not show statistically significant differences in the immediate post-treatment period. Grade 3 laboratory toxicity occurred in three patients (70–150 μm group, *n* = 2; 100–300 μm group, *n* = 1) with all of them experiencing elevated liver enzymes levels. However, a follow-up examination showed that the levels of the liver enzymes had normalized within 2 weeks. Child–Pugh score was elevated in six patients (Table 5). In four of the six patients, the Child-Pugh score was elevated by 1 point; the Child–Pugh score was elevated in three patients (A5→A6) due to a decrease in albumin level to <3.5 and in one patient (B8→B9) because of the presence of a small amount of new ascites on follow-up CT. The two patients whose Child–Pugh score increased by 2 points (B7→B9) were in the 100–300 μm group. One patient died of liver failure 4 months after DEB-TACE. The other patient has been living for >4 years with a Child–Pugh score of A6 after the liver function recovered.

## 4. Discussion

In the present study, there was no statistically significant difference in tumor response, post-embolization syndrome, or laboratory toxicity between 70–150 μm and 100–300 μm DEB-TACE. A few studies have evaluated the outcomes of these two types of DEB-TACE for patients with HCC [14,15,16,17]. In contrast to our results, Huo et al. showed that 70–150 μm DEB-TACE was associated with improved 1-month objective tumor response compared to 100–300 μm DEB-TACE (96.2% vs. 61.9%); however, both had a similar safety profile [15]. Another retrospective study compared the safety and efficacy of using one vial of 70–150 μm DEBs followed by one vial of 100–300 μm DEBs with two vials of 100–300 μm DEBs in TACE for HCC. The tumor response between the two groups was similar, but hepatobiliary adverse events occurred more frequently in the group using 70–150 μm DEBs than the group using 100–300 μm DEBs (25% vs. 9%) [14]. Two previous abstracts comparing 70–150 μm and 100–300 μm DEBs in TACE did not show any significant difference in tumor response between the two treatment groups [16,17]. One of the abstracts reported that grade 1 complications were more frequent in the 70–150 μm group than in the 100–300 μm group [16].

The tumor response achieved in the present study was comparable to that achieved in previous studies. In previous studies concerning DEB-TACE for HCC, 1–3 months of complete and objective response rates were 23.0–56.0% and 66.0–94.6%, respectively [13,20,21,22,23]. Different tumor response rates have been reported in studies, possibly due to differences in baseline patient and tumor characteristics. Tumor response can be affected by tumor size, tumor multiplicity, and Child–Pugh score [20,22,24]. One study using a 75-μm drug-eluting embolic agent showed an objective response rate of 66.0%, which is relatively low compared to the 91.4% objective response rate in the Korean multicenter registry, mainly using 100–300 μm DEBs [20,21]. However, the two studies differed significantly in the mean tumor size (5.8 cm vs. 3.6 cm) and Child–Pugh score (Child A: 68.8% vs. 94.1%) at baseline.

In the present study, 65% and 59.4% of patients in the 70–150 μm and 100–300 μm DEB groups, respectively, experienced at least one symptom of post-embolization syndrome (PES) after TACE; all the symptoms were mild and classified as grade 1 or 2. Among laboratory findings, transient elevation of liver enzymes and bilirubin levels was observed. PES can range from mild self-limited abdominal pain to severe symptoms; hence, the incidence of PES varies greatly in studies. Two previous studies on TACE with 70–150 μm DEBs reported PES rates of 65.1% and 100%, respectively [13,25]. However, there was a difference in the baseline tumor size and tumor vascularity in the two studies, and accordingly, there was a difference in the mean doxorubicin dose (31 mg vs. 59.7 mg). Although not statistically significant, in our study, the mean tumor size, mean doxorubicin dose, frequency of fever, and prolonged hospitalization due to persistent fever were higher in the 70–150 μm group than in the 100-300 μm group. Since 70–150 μm DEBs have a smaller particle size, to reach the same embolic endpoint for the same tumor, a larger volume of microspheres is required, which may result in a higher dose of doxorubicin used.

Hepatobiliary toxicity of DEB-TACE can be caused by local doxorubicin toxicity and ischemic injury of the peribiliary plexus, which has been a particular source of concern. Some authors have suggested that this type of toxicity may be more intense when small beads are used because small DEBs can penetrate the normal residual liver parenchyma more deeply [26,27,28]. Deipolyi et al. reported a higher rate of hepatobiliary adverse events in the group using 70–150 μm DEBs than in the group using 100–300 μm DEBs (25% vs. 9%) [14]. In our study, however, no biliary damage after the procedure was recorded, and significant deterioration of liver function (≥2 points in Child–Pugh score) was rare and exceptional. Recent studies on small DEB particles have reported biliary complication rates of 0%–6.8% and described most complications as asymptomatic [29,30]. Therefore, biliary toxicity may not be related to the DEB particle size; it may be related to the position of the microcatheter and the embolization endpoint [20,31].

There are several animal studies that compared 70–150 μm and 100–300 μm DEB-TACE. These animal studies have shown that 70–150 μm DEBs can penetrate more deeply and homogenously into the tumor, resulting in more intense ischemia and higher intratumoral doxorubicin concentrations [32,33,34]. In a previous study, only 42% of the occluded vessels were located inside the tumor when 100–300 μm DEB-TACE was performed [35]. Theoretically, TACE with 70–150 μm DEBs is supposed to have better curative effects than TACE with 100–300 μm DEBs. However, there were no significant differences between the two treatment modalities in the present study. Thus, more multi-center clinical trials with a larger sample size are required for further analysis.

This study has several limitations. First, this was a retrospective study with no long-term follow-up and survival data. However, we used multimodality treatments such as DEB-TACE, c-TACE, ablation therapy, or radiation if there was a recurrent or residual tumor after initial DEB-TACE. Therefore, long-term outcomes may not be representative of the treatment effect of first-line DEB-TACE. They can be affected by the multimodality treatment. Second, 70–150 μm DEBs were not available for use at our institution during the early study period. The 100–300 μm DEBs were chosen for TACE in the early study period, while 70–150 μm DEBs were used in the later study period. Although the operator was an experienced interventional radiologist, the embolization technique resulting from increasing experience with TACE can be reflected in the treatment outcome.

In conclusion, this retrospective study found no significant difference in tumor response between 70–150 μm and 100–300 μm DEB-TACE. Both groups showed favorable safety profiles, and the difference was not significant. Further prospective trials with large cohorts are needed to evaluate the optimal bead size to minimize adverse events and maximize the efficacy of DEB-TACE for HCC.

## Figures and Tables

**Table 1:** Baseline patient characteristics.

Characteristic	70–150 μm	100–300 μm	*p* Value
Numbers of patients	40	32	
Age	65.8 ± 11.2	62.2 ± 9.5	0.153
Male	28 (70.0%)	24 (75.0%)	0.638
Etiology			0.850
Hepatitis B	23 (57.5%)	20 (62.5%)	
Hepatitis C	6 (15.0%)	6 (18.8%)	
Alcohol	5 (12.5%)	3 (9.4%)	
Other	6 (15.0%)	6 (18.8%)	
Child–Pugh score			0.436
A	33 (82.5%)	24 (75.0%)	
B	7 (17.5%)	8 (25.0%)	
BCLC stage			0.965
0	6 (15.0%)	6 (18.8%)	
A	28 (70.0%)	21 (65.6%)	
B	5 (12.5%)	4 (12.5%)	
C	1 (2.5%)	1 (3.1%)	
Alfa-fetoprotein (AFP)			0.289
≤20 ng/mL	20 (50.0%)	20 (62.5%)	
>20 ng/mL	20 (50.0%)	12 (37.5%)	
Aspartate aminotransferase (AST)			0.267
≤45 U/L	24 (60.0%)	15 (46.9%)	
>45 U/L	16 (40.0%)	17 (53.1%)	
Alanine aminotransferase (ALT)			0.173
≤40 U/L	34 (85.0%)	23 (71.9%)	
>40 U/L	6 (15.0%)	9 (28.1%)	
Albumin			0.658
≤4 g/dL	27 (67.5%)	20 (62.5%)	
>4 g/dL	13 (32.5%)	12 (37.5%)	
Total bilirubin			0.097
≤1.2 mg/dL	31 (77.5%)	19 (59.4%)	
>1.2 mg/dL	9 (22.5%)	13 (40.6%)	
Prothrombin time			0.155
≤1.2 INR	23 (57.5%)	13 (40.6%)	
>1.2 INR	17 (42.5%)	19 (59.4%)	

BCLC, Barcelona Clinic Liver Cancer; INR, international normalized ratio. Note: Data are expressed as n (%) or mean ± standard deviation. The BCLC staging system modified by AASLD guidance 2018 is used.

**Table 2:** Details of tumor characteristics and DEB-TACE.

Characteristic	70–150 μm	100–300 μm	*p* Value
Numbers of patients	40	32	
Tumor distribution			0.624
Single	26 (65.0%)	19 (59.4%)	
Multiple	14 (35.0%)	13 (40.6%)	
Tumor location			0.453
Unilobar	37 (92.5%)	27 (84.4%)	
Bilobar	3 (7.5%)	5 (15.6%)	
Maximum tumor diameter (cm)	3.57 ± 2.2	2.99 ± 1.47	0.185
Sum of tumor diameters (cm)	4.45 ± 3.26	3.91 ± 2.4	0.437
Dose of doxorubicin (mg)	28.8 ± 14.2	23.2 ± 12.5	0.083
Selective catheterization			0.123
Segmental	14 (35.0%)	17 (53.1%)	
Subsegmental	26 (65.0%)	15 (46.9%)	
Additional bland embolization	3 (7.5%)	2 (6.3%)	1.000

Note: Data are expressed as *n* (%) or mean ± standard deviation.

**Table 3:** Tumor response (mRECIST) after DEB-TACE.

Response	70–150 μm (*n* = 40)	100–300 μm (*n* = 32)	*p* Value
Complete response	19 (47.5%)	11 (34.4%)	0.262
Partial response	15 (37.5%)	15 (46.9%)	0.423
Objective response	34 (85.0%)	26 (81.3%)	0.671
Stable disease	4 (10.0%)	5 (15.6%)	0.498
Progressive disease	2 (5.0%)	1 (3.1%)	1.000

DEB-TACE, drug-eluting bead transcatheter arterial chemoembolization; mRECIST, modified response evaluation criteria in solid tumors. Note: Data are expressed as *n* (%).

**Table 4:** Comparison of tumors, patients, and procedural characteristics according to the complete response.

Characteristics	Complete Response *n* = 30	No Complete Response *n* = 42	*p* Value
Tumor distribution			0.267
Single	21 (70%)	24 (57.1%)	
Multiple	9 (30%)	18 (42.9%)	
Tumor location			0.128
Unilobar	29 (96.7%)	35 (83.3%)	
Bilobar	1 (3.3%)	7 (16.7%)	
Maximum tumor diameter (cm)	2.68 ± 1.46	3.77 ± 2.09	0.011
Sum of tumor diameters (cm)	3.17 ± 1.79	4.96 ± 3.31	0.004
AFP			0.873
≤20 ng/mL	17 (56.7%)	23 (54.8%)	
>20 ng/mL	13 (43.3%)	19 (45.2%)	
Child–Pugh score			0.185
A	26 (86.7%)	31 (73.8%)	
B	4 (13.3%)	11 (26.2%)	
BCLC stage			0.131
0	8 (26.7%)	4 (9.5%)	
A	20 (66.7%)	29 (69%)	
B	2 (6.7%)	7 (16.7%)	
C	0 (0%)	2 (4.8%)	
Dose of doxorubicin (mg)	24.2 ± 11.2	27.8 ± 15.1	0.251
Selective catheterization			0.658
Segmental	12 (40%)	19 (45.2%)	
Subsegmental	18 (60%)	23 (54.8%)	
Additional bland embolization	2 (6.7%)	3 (7.1%)	1

Note: Data are expressed as *n* (%) or mean ± standard deviation.

**Table 5:** Incidences of clinical adverse events after DEB-TACE.

Adverse Events	70–150 μm (*n* = 40)	100–300 μm (*n* = 32)	*p* Value
Abdominal pain	17 (42.5%)	14 (43.8%)	0.981
Grade 1/2/3	8/9/0	7/7/0	
Vomiting	3 (7.5%)	2 (6.3%)	1.000
Grade 1/2/3	3/0/0	2/0/0	
Fever	21 (52.5%)	11 (34.4%)	0.124
Grade 1/2/3	21/0/0	11/0/0	
Prolonged hospitalization (≥7 days)	4 (10.0%)	1 (3.1%)	0.373
Increased Child–Pugh score after 1 month	1 (2.5%)	5 (15.6%)	0.082
A5→A6	1	2	
B7→B9	0	2	
B8→B9	0	1	

Note: Data are expressed as *n* (%).

**Table 6:** Laboratory changes after DEB-TACE.

Variable	70–150 μm (*n* = 40)	100–300 μm (*n* = 32)	*p* Value
AST	37 (14.5–74.5)	32 (6–65)	0.389
ALT	28 (3.5–55.5)	16 (3–39)	0.316
Albumin	−0.3 (−0.6–−0.1)	−0.3 (−0.5–0.0)	0.419
Total bilirubin	0.54 (0.28–0.69)	0.56 (0.30–0.81)	0.586
Prothrombin time	0.12 (0.06–0.21)	0.10 (0.04–0.18)	0.234

ALT, alanine aminotransferase; AST, aspartate aminotransferase; DEB-TACE, drug-eluting bead transarterial chemoembolization. Note: Data are expressed as median (25th–75th percentile).

## Data Availability

Not applicable.
